# Investigating Reddit Data on Type 2 Diabetes Management During the COVID-19 Pandemic Using Latent Dirichlet Allocation Topic Modeling and Valence Aware Dictionary for Sentiment Reasoning Analysis: Content Analysis

**DOI:** 10.2196/51154

**Published:** 2025-02-21

**Authors:** Meghan Nagpal, Niloofar Jalali, Diana Sherifali, Plinio Morita, Joseph A Cafazzo

**Affiliations:** 1 Institute of Health Policy, Management, & Evaluation, Dalla Lana School of Public Health, University of Toronto, Health Sciences Building, 4th Floor, Toronto, ON, M5T 3M6, Canada, 1 416 978 4326, 1 416 978 7350; 2 Centre for Digital Therapeutics, Techna Institute, University Health Network, Toronto, ON, Canada; 3 School of Public Health & Health Systems, University of Waterloo, Kitchener, ON, Canada; 4 School of Nursing, McMaster University, Hamilton, ON, Canada; 5 Department of Computer Science, University of Toronto, Toronto, ON, Canada; 6 Institute of Biomedical Engineering, Faculty of Applied Science & Engineering, University of Toronto, Toronto, ON, Canada

**Keywords:** diabetes, diabetes mellitus, DM, COVID-19, pandemics, social media, health behavior, health knowledge, attitudes, practice, self-management, patient-generated health data, perspective, T2DM

## Abstract

**Background:**

Type 2 diabetes (T2D) is a chronic disease that can be partially managed through healthy behaviors. However, the COVID-19 pandemic impacted how people managed T2D due to work and school closures and social isolation. Moreover, individuals with T2D were at increased risk of complications from COVID-19 and experienced worsened mental health due to stress and anxiety.

**Objective:**

This study aims to synthesize emerging themes related to the health behaviors of people living with T2D, and how they were affected during the early stages of the COVID-19 pandemic by examining Reddit forums dedicated to people living with T2D.

**Methods:**

Data from Reddit forums related to T2D, from January 2018 to early March 2021, were downloaded using the Pushshift API; support vector machines were used to classify whether a post was made in the context of the pandemic. Latent Dirichlet allocation topic modelling was performed to identify topics of discussion across the entire dataset and a subsequent iteration was performed to identify topics specific to the COVID-19 pandemic. Sentiment analysis using the VADER (Valence Aware Dictionary for Sentiment Reasoning) algorithm was performed to assess attitudes towards the pandemic.

**Results:**

From all posts, the identified topics of discussion were classified into the following themes: managing lifestyle (sentiment score 0.25, 95% CI 0.25-0.26), managing blood glucose (sentiment score 0.19, 95% CI 0.18-0.19), obtaining diabetes care (sentiment score 0.19, 95% CI 0.18-0.20), and coping and receiving support (sentiment score 0.34, 95% CI 0.33-0.35). Among the COVID-19–specific posts, the topics of discussion were coping with poor mental health (sentiment score 0.04, 95% CI −0.01 to0.11), accessing doctor and medications and controlling blood glucose (sentiment score 0.14, 95% CI 0.09-0.20), changing food habits during the pandemic (sentiment score 0.25, 95% CI 0.20-0.31), impact of stress on blood glucose levels (sentiment score 0.03, 95% CI −0.03 to 0.08), changing status of employment and insurance (sentiment score 0.17, 95% CI 0.13-0.22), and risk of COVID-19 complications (sentiment score 0.09, 95% CI 0.03-0.14). Overall, posts classified as COVID-19–related (0.12, 95% CI 0.01-0.15) were associated with a lower sentiment score than those classified as nonCOVID (0.25, 95% CI 0.24-0.25). This study was limited due to the lack of a method for assessing the demographics of users and verifying whether users had T2D.

**Conclusions:**

Themes identified from Reddit data suggested that the COVID-19 pandemic significantly influenced how people with T2D managed their disease, particularly in terms of accessing care and dealing with the complications of the virus. Overall, the early stages of the pandemic negatively impacted the attitudes of people living with T2D. This study demonstrates that social media data can be a qualitative data source for understanding patient perspectives.

## Introduction

### Background

Diabetes is a serious metabolic condition in which the body experiences elevated blood glucose levels, which can result in serious complications such as cardiovascular disease, kidney disease, stroke, eye disease, foot ulcers, nerve damage, and amputation. The World Health Organization [1] states that high blood glucose levels are the third leading cause of premature mortality. Type 2 diabetes (T2D) is characterized by insulin resistance or insufficient production of insulin. Research suggests that the risks of complications in people living with T2D can be mitigated through self-management, including glucose levels monitoring, optimized nutrition, regular physical activity, and taking prescribed medications [2,3].

### Managing T2D During the COVID-19 Pandemic

With the emergence of the COVID-19 pandemic in March 2020, nearly the entire global population was affected by social distancing measures that included business closures, remote schooling and work, prohibition of large crowds, limited socialization, and virtual outpatient diabetes care delivery. Beyond the fear and anxiety that manifested [4], individuals with T2D were at an increased risk of complications from COVID-19, as the virus thrives in an environment of high blood glucose [5]. T2D was considered among the most prevalent chronic conditions where patients were at serious risk of hospitalization or death if they contracted the virus [6]. This was primarily due to increased inflammation from obesity and insulin resistance, and prevalence of other comorbidities such as hypertension, cardiovascular disease, dyslipidemia, and older age [7]. Because T2D is mostly managed by lifestyle behaviors, COVID-19 restriction measures reduced physical activity and increased other unhealthy behaviors among those living with T2D and other chronic conditions [4,8]. Moreover, there was a concern about limited access to health care for people with T2D, as telemedicine replaced many in-person visits [9]. Overall, people with T2D exhibited worsening glucose levels during lockdown periods [10].

### Rationale and Study Objectives

Given that the pandemic impacted the lifestyle behaviors of individuals with T2D, it is reasonable to assume that there were changes in how they managed their condition, which contributed to added stress and anxiety. Many people with T2D use social medial forums to discuss how they manage their condition through sharing information on diet, symptoms, research findings, and recipes, while seeking peer support [11]. Social media also serves as a public data source to gauge sentiment and topics of discussion during the initial lockdown period. The objectives of this study were to synthesize emerging themes from peer discussion on Reddit, a social media forum with dedicated communities for individuals coping with T2D. Using data harnessed through the Pushshift application programming interface (API), this study aimed to understand how their attitudes and diabetes management evolved during the early stages of the pandemic. Reddit was chosen over other social networking platforms due to the segregation of special interest communities, such as those focused on diabetes, and the availability of the Pushshift API as an open-source tool for data scraping.

## Methods

### Overview

Support vector machines were used to classify Reddit posts related to the COVID-19 pandemic. Latent Dirichlet allocation (LDA) topic modeling [12] and sentiment analysis using the VADER (Valence Aware Dictionary for Sentiment Reasoning) algorithm [13] were performed to achieve the study objectives. Algorithms were executed through Python scripts in the Jupyter Notebook platform version 6 (Linux Foundation), with final extractions outputted as CSV files and further analyzed through Microsoft Excel. No predefined protocol was used for this experiment, given the novelty of social media analysis at the time of the study.

### Data Source and Collection

For this study, 3 communities on Reddit were examined: r/type2diabetes, r/diabetes_t2, and r/diabetes [14-16]. From the r/diabetes community, only posts tagged with the “flair” and “type 2 diabetes” were included. Raw data of 100,887 posts between January 1, 2018, to March 5, 2021, from these 3 communities were collected from Reddit in March 2021 via the Pushshift application programming interface (API) using Python scripts in Jupyter Notebook [17]. Data was exported as CSV files and examined in Microsoft Excel. Posts with less than 5 words, posts with the label “removed” and those containing special characters were removed from the dataset using Microsoft Excel filtering tools to avoid bias in the algorithm.

### Classification of Posts

Manual examination of the dataset found that the first post about the COVID-19 pandemic was written on February 28, 2020, and coincidentally, the last post about the pandemic in the dataset was made on February 28, 2021. In total, 48,988 posts were published during this period. Within the dataset, posts were manually searched for terms related to the COVID-19 pandemic . These terms included “COVID”, “coronavirus”, “pandemic”, “social distancing”, “lockdown”, “quarantine”, “toilet paper”, “unemploy” and “unemployed”, “work and working from home”, “telehealth”, “vaccine”, “sanitizer”, and “mask.”

Posts that contained these terms in the text body were manually evaluated for context and labeled as COVID or nonCOVID. In total, 9803 posts were manually classified, of which 2065 were labeled as COVID and 7738 as nonCOVID; these posts were subsequently classified using support vector machines. An additional 818 posts made in the context of the COVID-19 pandemic were identified, bringing the total number of pandemic-specific posts to 2883. The remaining unclassified posts from the identified pandemic period were labeled as nonCOVID.

### Data Analysis

The LDA topic modeling algorithm [18] was applied in two iterations to identify topics of discussion by obtaining clustering words belonging to a single topic. A total of 85,266 posts analyzed, with 2682 being specific to the COVID-19 pandemic. The first iteration of topic modeling was performed on the entire processed dataset of 85,266 posts made between January 1, 2018, and March 5, 2021. The second iteration was performed on a subset of this dataset comprising the 2682 posts specifically related to the COVID-19 pandemic.

Sentiment analysis was performed to understand the subjective emotions or sentiment associated with each post, using a normalized compound score between −1 and 1. The compound score thresholds for classifying text into 3 sentiment categories were as per the VADER algorithm [19]: positive (≥0.05), neutral (between −0.05 and 0.05), and negative (≤−0.05). Further statistical analysis was performed with R software version 4 (R Foundation for Statistical Computing), to calculate mean sentiment scores and 95% CI.

### Ethical Considerations

According to the University of Toronto’s research ethics guidelines, ethical approval was not sought for this study as per exemptions in Section 1, as Reddit was assumed to be a public data source with no reasonable expectation of privacy [20]. Moreover, there was no direct interaction between the researchers and participants; therefore, ethical approval was not necessary, as per Section 2 of the guidelines [20]. The data used for qualitative analysis were scraped directly from Reddit. As users do not need authentication to view Reddit forums, it was assumed that the users who made these posts were aware that they would be displayed publicly. As this is a publicly available dataset, participants were not compensated.

Data for this study was collected through the Pushshift API. Over 100 published research studies have already used Reddit data extracted through this API [21]. Proferes et al [22] conducted a systematic analysis of 727 manuscripts that used Reddit as a data source and found that fewer than 15% mentioned undergoing any form of ethical review.

While the usernames of the post authors’ were obtained, they were assumed to be pseudonyms and not their actual names. However, we acknowledge that some users may have integrated their real names into their usernames; in this study, the usernames were removed to deidentify the data. Reddit does not provide identifiable characteristics of individual users, such as their names, genders, or geographical locations. However, it is possible that some users may include identifiable information within their posts. In this study, such identifiable information was not extracted or analyzed. Since the posts could not be traced to any individual, no compensation was provided.

## Results

### Topics of Discussion

**Figure 1.**
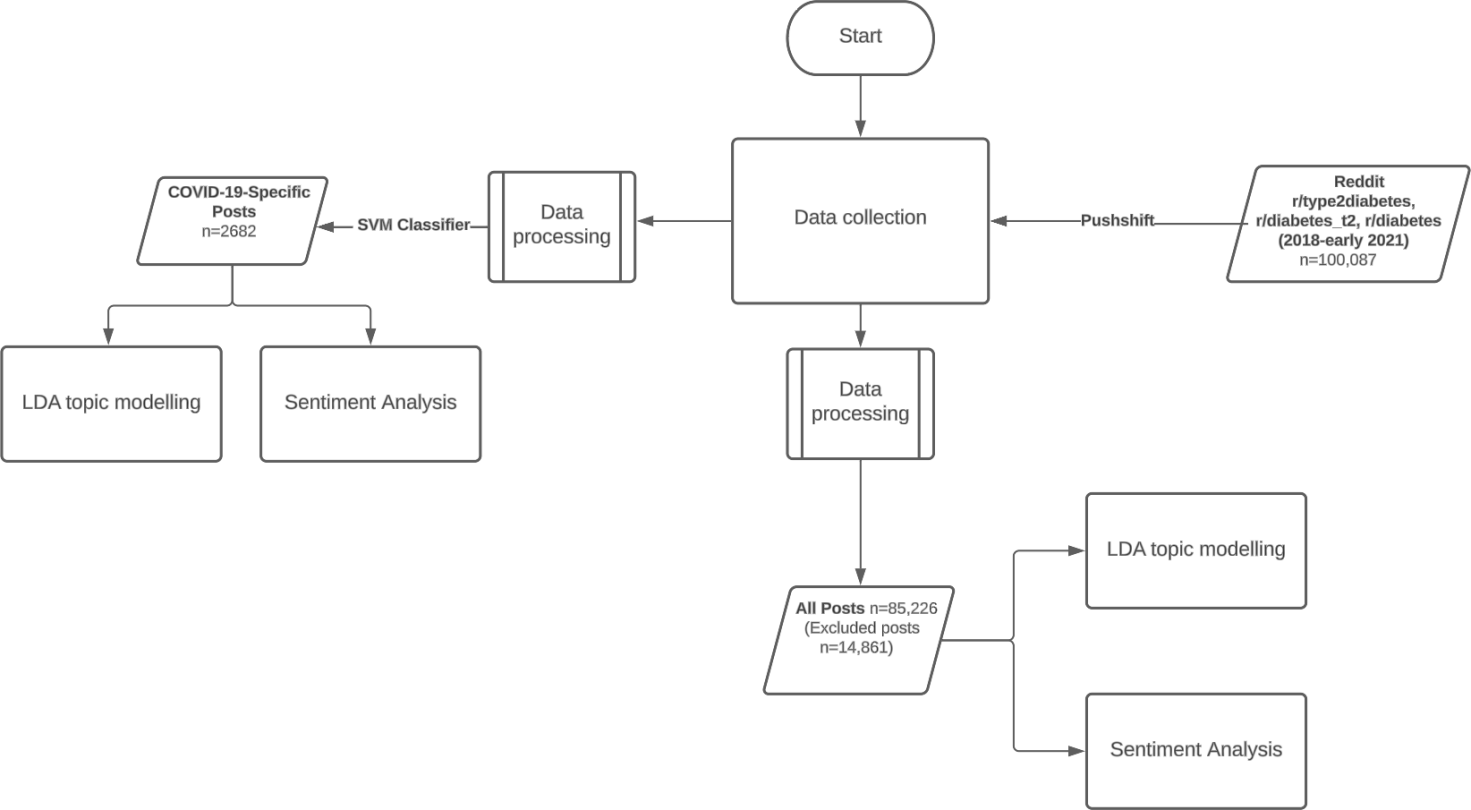
Study methodology conducted in early 2021. LDA: latent Dirichlet allocation; SVM: support vector machine.

The final processed dataset consisted of 85,266 posts made between January 1, 2018, and March 3, 2021. The study methodology is shown in Figure 1. Data were collected from Reddit diabetes communities through the Pushshift API and classified as COVID or nonCOVID. LDA topic modelling and sentiment analysis was conducted on processed text. The topics were manually labeled and categorized into broader themes as shown in Table 1.

**Table 1.** Words extracted from topic modeling of processed data of all Reddit posts in diabetes communities between January 2018–March 2021.

Themes and topic names	Words
Theme 1: Managing blood glucose	
Using blood glucose meters	meter, insurance, reading, check, strip, libre, sensor, finger, cover, cgm, free, pay, app, stick, test, track, buy, freestyle, monitor, difference
Controlling blood glucose	glucose, blood, high, glucose, level, normal, low, range, control, number, spike, check, average, mgdl, raise, point, fast, affect, measure, test
Controlling HbA_1C_ levels	ac, month, weight, year, lose, start, med, diagnose, metformin, week, diet, stop, lb, change, medication, drop, loss, exercise, pound, control
Rationale for glucose variability	insulin, type, body, resistance, gt, disease, people, medication, risk, cure, increase, reverse, cell, case, pancreas, term, treatment, long, produce, diabetic
Theme 2: Managing lifestyle	
Exercising for diabetes care	work, time, exercise, walk, bit, long, pretty, day, start, minute, run, ill, thing, week, stress, home, stay, big, lot, hit
Timing meals and snacks	fast, hour, meal, bg, metformin, morning, time, number, week, mg, night, eat, effect, day, start, low, dinner, breakfast, sleep, reading
Eating low carbohydrate or ketogenic diet	diet, low, keto, carb, fat, body, protein, calorie, lot, liver, carbs, healthy, process, cholesterol, reduce, energy, high, carbohydrate, intake, muscle
Controlling carbohydrates and alcohol	eat, carbs, food, drink, water, cut, meal, carb, avoid, lot, spike, soda, fiber, drinking, limit, small, alcohol, gram, sweet, ate
Options for food	bread, rice, add, cheese, egg, potato, meat, cream, fruit, veggie, chicken, taste, pasta, recipe, snack, butter, nut, coffee, salad, milk
Theme 3: Coping and receiving support	
Attitudes towards disease	change, life, great, lot, thing, work, dr, make, learn, hope, luck, live, easy, control, love, healthy, manage, hard, lifestyle, sound
Caregiving for family and history	feel, time, family, year, felt, thought, happen, care, make, deal, mom, friend, turn, kind, super, shit, die, guess, hard, suck
Theme 4: Obtaining diabetes care	
Interacting with care team	doctor, test, result, advice, talk, doc, give, diagnose, endo, read, appointment, wait, endocrinologist, follow, diagnosis, time, thought, adjust, mention, idea
Educating for self-management	people, post, health, question, medical, understand, research, information, study, read, answer, agree, link, support, comment, patient, diabetic, base, opinion, group
Managing complications/comorbidities	issue, problem, symptom, pain, foot, effect, bad, eye, year, damage, heart, experience, kidney, stomach, hand, stop, happen, shot, neuropathy, vision

A subset of 2682 posts written between February 28, 2020, and February 28, 2021, was classified as COVID-specific. The topics were manually labeled as shown in Table 2.

**Table 2.** Words extracted from topic modeling of processed data from all COVID–related Reddit posts in diabetes communities from 28 February 2020 to 28 February 2021.

Topic Name	Words
Coping with poor mental health	feel, day, week, work, weight, lose, walk, bit, symptom, bad, lot, gym, night, happen, end, great, covid, felt, ill, diagnosis
Accessing doctor and medications and controlling blood glucose	doctor, test, ac, year, insulin, month, metformin, diagnose, low, glucose, diet, week, med, exercise, change, level, stop, reading, check, medication
Changing food habits during the pandemic	eat, food, carbs, lot, meal, low, carb, diet, hard, hour, thing, water, keto, fast, cut, easy, glucose, add, stuff, rice
Impact of stress on blood glucose levels	blood, glucose, high, time, stress, number, long, exercise, bg, body, problem, morning, normal, control, sleep, make, level, effect, change, kind
Changing status of employment and insurance	work, home, hospital, today, time, year, talk, give, advice, wait, insurance, visit, guess, state, order, meter, live, strip, situation, friend
Risk of COVID complications	covid, type, health, care, risk, sick, control, issue, virus, disease, diabetic, question, pandemic, mask, hand, case, infection, wear, patient, home

### Sentiment Analysis

Using the VADER algorithm, the compound sentiment score of each post was determined. The sentiment across all posts in the dataset was positive with a mean compound sentiment score of 0.23 (95% CI 0.23-0.24).

### Comparison of Sentiment of COVID Versus NonCOVID Posts

The sentiment was compared between posts that were classified as COVID and nonCOVID. The mean compound scores of COVID-related posts remained within the positive threshold (sentiment score 0.12, 95% CI 0.01-0.15) but was lower than that of nonCOVID-related posts (sentiment score 0.25, 95% CI 0.24-0.25).

### Sentiment by Theme

**Figure 2.**
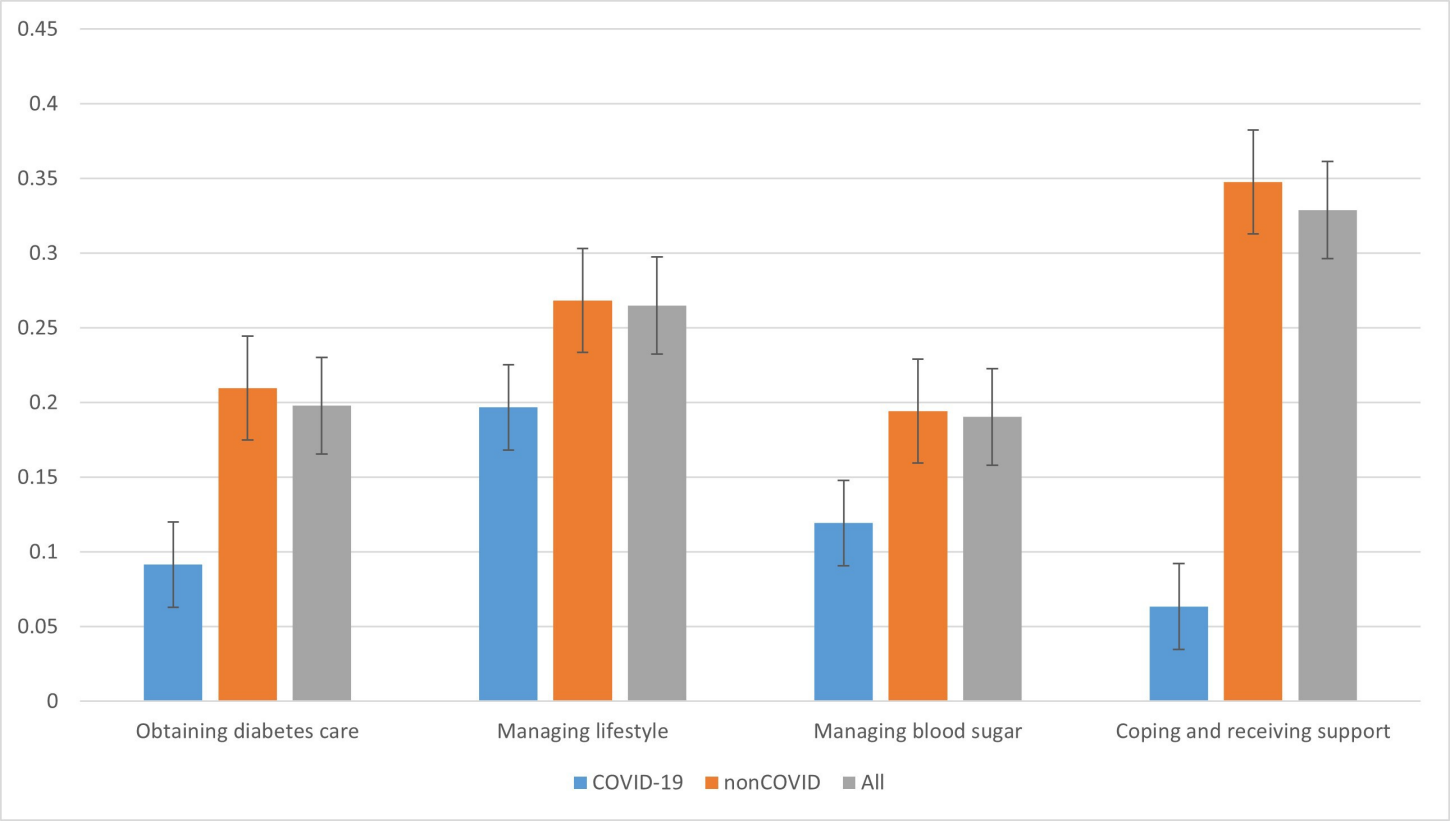
Mean compound sentiment score of all posts, sorted by theme and classified as covid-related, noncovid-related, or combined.

Coping and receiving support had the highest mean compound score (sentiment score 0.34, 95% CI 0.33-0.35), followed by managing lifestyle (sentiment score 0.25, 95% CI 0.25-0.26), obtaining diabetes care (sentiment score 0.19, 95% CI 0.18‐0.20), and managing blood glucose (0.19, 95% CI 0.18‐0.19) (Figure 2). All mean compound scores fall in the threshold to be classified as positive, with coping and receiving support demonstrating the strongest positive intensity score.

### Sentiment by Theme Amongst COVID-Classified and NonCOVID Posts

Mean compound scores were further analyzed among posts classified as COVID-related or as nonCOVID-related. Coping and receiving support had the highest mean compound score (sentiment score 0.35, 95% CI 0.33‐0.36), followed by managing lifestyle (sentiment score 0.27, 95% CI 0.26‐0.28), obtaining diabetes care (sentiment score 0.21, 95% CI 0.20‐0.22), and managing blood glucose (0.19, 95% CI 0.18‐0.20). However, for COVID-related posts, coping and receiving support had the lowest mean compound score (sentiment score 0.06, 95% CI −0.02 to 0.13), which was slightly above the threshold for positive classification. Ranked from lowest to highest, obtaining diabetes care had the second-lowest mean compound score (sentiment score 0.09, 95% CI 0.05‐0.13), followed by managing blood glucose (sentiment score 0.12, 95% CI 0.08‐0.16), and managing lifestyle (sentiment score 0.20, 95% CI 0.15‐0.24).

### Sentiment by COVID-Specific Topics

Sentiment was further analyzed across the topics generated for the COVID-related posts (Table 3).

**Table 3.** Mean compound sentiment score of COVID-related posts sorted by topic.

Topic	Compound sentiment score, mean (95% CI)
Accessing doctor and medications and controlling blood sugar	0.14 (0.09‐0.20)
Changing status of employment and insurance	0.17 (0.13‐0.22)
Changing food habits during the pandemic	0.25 (0.20‐0.31)
Coping with poor mental health	0.04 (−0.01 to 0.11)
Risk of COVID-19 complications	0.09 (0.03‐0.14)
Impact of stress on blood glucose levels	0.03 (–0.03 to 0.08)

Impact of stress on blood glucose levels and coping with poor mental health had mean compound scores that were within the threshold of being classified as neutral (sentiment score 0.03, 95% CI −0.03 to 0.08 and 0.04, 95% CI −0.01 to 0.11, respectively). These were followed by risk 0f COVID complications (sentiment score 0.09, 95% CI 0.03‐0.14), accessing doctor and medications and controlling blood glucose (sentiment score 0.14, 95% CI 0.09‐0.20), and changing status of employment and insurance (sentiment score 0.17, 95% CI 0.13‐0.22), ranked from lowest to highest. Finally, changing food habits had the highest mean compound score (sentiment score 0.25, 95% CI 0.20‐0.31).

## Discussion

Our study confirmed that T2D subreddits were used for peer support to discuss themes related to diabetes management in depth. Oyebode and Orji [23] and Griffis et al [24] found that the themes for discussion of diabetes management on online platforms focused on nutrition, lifestyle, symptoms, treatments, research, risk factors, and supplements or remedies. Additionally, we found that topics of discussion also included glycemic control and support-seeking behaviors. The overall emerging themes identified in this study include health behaviors such as glycemic control, exercise, and nutrition, along with attitudes towards diabetes, interactions with family and caregivers, management of comorbidities, and educational resources. Topics of discussion were specific to the COVID-19 pandemic and were generally associated with lower sentiment, with specific discussions focusing on glycemic and lifestyle management under unique circumstances, including changing food habits, increased stress, deteriorated mental health, difficulties in accessing health care, and fear of complications due to COVID-19. Additional themes of discussion included access to diabetes care during the pandemic and the impact on employment and insurance during this period.

### Topics Discussed on Reddit Communities

Topics discussed on Reddit communities for people managing T2D included glycemic control, exercise, and nutrition, indicating that users were discussing health behaviors. Additionally, users discussed their attitudes toward their disease, interactions with their families and caregivers, management of comorbidities, and shared educational resources. This indicated that online forums provide opportunities for holistic discussions that consider the person as a whole, including the social and psychological factors impacting the management of diabetes. The possibilities for such discussions are endless, which may not be captured in structured clinical settings.

### COVID-Specific Discussions on Reddit Forums

Topics extracted from COVID-specific posts were about accessing medical care, the impact of stress on glycemic control, changing food habits, poor mental health, and fear of complications due to COVID. This was in line with a literature review of conventional qualitative studies that assessed the impact of the pandemic on people with T2D [8]. Additionally, we found that the changing status of employment and insurance status was also a topic of discussion among the COVID-specific posts. These data supports our finding that discussions among people with T2D on Reddit offer insights from a holistic perspective, considering various aspects of a person’s life related to their disease.

### Sentiment Analysis

For all posts in the dataset, the mean compound sentiment score as per the VADER algorithm was 0.23 (95% CI, 0.23‐0.24). As this score is above the threshold of 0.05, we conclude that, on an average, posts in the Reddit communities for people with diabetes were positive.

A significant difference in sentiment was observed between the COVID-related and nonCOVID posts. Overall, the mean compound score of posts labeled as “nonCOVID” was 0.24 (95% CI 0.24‐0.25), while posts labeled as COVID had a mean compound score of 0.12 (95% CI 0.01‐0.15), which was significantly lower. While we cannot conclude that the COVID-labeled posts were overall negative in tone, their sentiment intensity was lower.

Among the nonCOVID posts, coping and receiving support was associated with the highest mean compound score (sentiment score 0.35, 95% CI 0.33‐0.36). However, for COVID-related posts, coping and receiving support had the lowest mean compound score (sentiment score 0.06, 95% CI −0.01 to 0.13), slightly above the threshold to be classified as positive. This indicated that the pandemic negatively impacted people with T2D.

While specifically analyzing the topics generated only among the COVID-related posts, changing food habits (sentiment score 0.25, 95% CI 0.20‐0.31) had the highest mean compound score, indicating that even though eating patterns were impacted during the pandemic, there was peer support and camaraderie in discussions about food choices. Conversely, the impact of stress on blood glucose levels and coping with poor mental health had the lowest mean compound scores (sentiment score 0.03, 95% CI −0.03 to 0.08 and 0.05, 95% CI −0.01 to 0.11, respectively). These low sentiment scores suggest that the pandemic resulted in anxiety that affected glycemic control and resulted in poorer mental health outcomes.

### Impact of the Pandemic on Managing T2D

Our study identified key themes for managing T2D during the pandemic from Reddit communities and the associated sentiment toward these themes.

#### Access to Diabetes Care During the Pandemic

During the COVID-19 pandemic, accessing care was perceived as a significant barrier to managing T2D, as more clinical visits were through web-based platforms. Khader, Jabeen, and Namoju [25] found that in a study of 1582 participants in India, the frequency of clinical visits was reduced in 87.28% of participants, and 87.81% of participants did not have access to health services. Al-Sofiani et al [26] found that among 568 participants in the Arab Gulf region, a lack of communication with the health care providers was associated with higher odds of depression and anxiety among people with diabetes. These findings were further confirmed through our sentiment analysis. Among all posts, the mean compound sentiment score for posts related to the theme of obtaining diabetes care was 0.19 (95% CI 0.18‐0.20); however, among the COVID-specific posts, this score significantly dropped to 0.09 (95% CI 0.05‐0.13).

Further, topic modeling among COVID-specific posts also identified access to doctor and controlling blood glucose as a topic for discussion. While barriers to accessing health care providers existed, another barrier including the fear of acquiring a COVID-19 infection and avoiding hospitalization in potentially dangerous situations was identified. Further research should examine the impact of reduced in-person health care visits among people with T2D.

#### Impact on Employment During the Pandemic

Efforts to curb the spread of the virus resulted in employers requiring employees to work from home [27]. As this was a disruption to daily life, Reddit discussions suggested that this disruption impacted the health behaviors of individuals managing T2D.

In addition to work-from-home measures, the pandemic also resulted in the loss of employment due to the shutting down of businesses to curb the spread of the virus. Unemployment not only affected income and standard of living but also decreased their sense of purpose [28], potentially impacting health behaviors due to increased stress. Furthermore, many unemployed people were also impacted by changes to their health insurance caused by job loss [29,30]. From Reddit discussions, it could be inferred that loss of insurance created barriers to obtaining medications, blood glucose meters, and test strips, ultimately affecting glycemic control for people with T2D.

#### Glycemic Control During the Pandemic

Managing blood glucose was a major theme for discussion across our dataset. Glycemic control is a major component of self-management of T2D and was expected to be a major theme of discussion among peers. Our analysis found that specifically during the pandemic, users of the Reddit communities for managing T2D reported that the pandemic-related stress affected their blood glucose levels. Laboratory studies have demonstrated that psychological stressors are linked to hyperglycemia [31-33].

Overall, managing blood glucose was the theme with the lowest mean sentiment score (0.19, 95% CI 0.18‐0.19), which was further reduced among the COVID-related posts (0.12, 95% CI 0.08‐0.16). This correlation suggested that attitudes toward managing blood glucose are associated with lower sentiment compared to other themes and that the pandemic further increased concerns about glycemic management. Specifically, the impact of stress on blood glucose levels had the lowest sentiment score (0.03, 95% CI −0.03 to 0.08), suggesting that users had increased anxiety about glycemic control.

Additionally, the topic associated with accessing a doctor also included terms related to controlling blood glucose. This suggested that reduced access to a health care provider may have affected glycemic control in people with T2D through limited A1c monitoring or medication access during the pandemic. Changes to employment were associated with reduced access to health insurance, which further resulted in reduced access to medications, blood glucose meters, and test strips, affecting glycemic control.

#### Lifestyle Management During the Pandemic

In our dataset, lifestyle management was the most frequent theme of discussion, with a positive mean sentiment score of 0.25 (95% CI 0.25‐0.26). Among the COVID-specific posts, this sentiment score dropped to 0.20 (95% CI 0.15‐0.24) but was overall positive. Changing food habits during the pandemic was associated with the highest mean sentiment score (0.25, 95% CI 0.20‐0.31), indicating that increased unhealthy food consumption may have been a coping mechanism associated with positive emotions through the stressful time.

Interestingly, the terms “gym” and “walk” were included among the COVID-specific posts under the topic coping with poor mental health. This suggests that reduced physical activity was attributed to gym closures in the initial months of the pandemic and people relied on walking as a means of physical activity. Furthermore, physical activity is also another means of coping with stressors and many people were unable to be physically active while staying at home. Further investigation by clinicians is needed to help people with T2D cope during changes such as pandemic-related lockdowns, so that they could continue to maintain a healthy lifestyle during stressful times.

#### Mental Health During the Pandemic

The mental health impact of this pandemic were expected to be long-term due to the extreme measures necessary to prevent the spread of the virus and the resulting economic implications [34]; people with T2D were no exceptions to this. In our study, we found that while coping and receiving support was associated with the highest mean sentiment overall in our dataset (sentiment score 0.33, 95% CI 0.33‐0.35), among the COVID-19–related posts, it was associated with the lowest mean sentiment (sentiment score 0.06, 95% CI −0.01 to 0.13). This finding suggests that while Reddit forums for managing T2D have been helpful overall for people with the disease, the pandemic negatively impacted how people coped and their attitudes toward their disease. Specifically, among the COVID-19–related posts, discussions about coping with poor mental health and risk of COVID complications were associated with lower mean sentiment scores (0.05, 95% CI −0.01 to 0.11 and 0.09, 95% CI 0.03-0.14, respectively). Although our study demonstrated that people with T2D used peer support to cope with the stressors of the pandemic, it also highlights the negative impact caused by these psychological stressors. Moreover, the implications of potentially being exposed to the virus posed an additional source of anxiety, given the risk of serious COVID-19 complications among people with T2D. As discussed in this paper, the implications of poor mental health and anxiety regarding COVID-19 exposure affected glycemic control and lifestyle management, both of which are necessary to prevent complications from diabetes. Furthermore, the anxiety related to COVID-19 exposure served as a barrier to seeking care at urgent care centres and hospitals when necessary.

### Summary of Findings

This study builds upon the work of Nagpal et al [11], which suggests that public social media forums can serve as a form of patient-generated health data for individuals with chronic conditions such as T2D. Social media forums such as Reddit provide a platform for individuals with chronic diseases to discuss their condition, particularly during times of collective chaos, such as the global COVID-19 pandemic; the insights generated from these discussions provide a valuable source of qualitative knowledge. Consistent with the findings from conventional qualitative studies, this study found that health behaviors of people with T2D were impacted during the pandemic, along with declines in mental health [8], as reflected in the sentiment scores. In particular, the pandemic raised concerns about COVID-19 exposure and access to health care. However, our analysis also indicated that employment and insurance were impacted during the pandemic, affecting how people managed their diabetes care. This finding suggests that social media data can be a valuable source of qualitative data to capture health behaviors during particular periods, such as the global COVID-19 pandemic. As the world continues to evolve due to geopolitics, wars, diseases, and climate change, social media can be a source of qualitative data to gauge attitudes toward chronic disease management.

### Limitations of the Study

While topic modeling and sentiment analysis were performed on the dataset, researchers did not manually examine each post for context and cannot comment on the quality of discussions within the forum. Additionally, we cannot confirm that all users who contributed posts were people living with T2D. This study only examined 3 Reddit communities mentioned earlier, and did not include other subreddits related to COVID-19 or mental health.

Furthermore, there is no information about the demographics of users specific to the diabetes forums of Reddit, and we assumed that their demographics were similar to those of general Reddit users. Under this assumption, there may be a sampling bias in our findings, as Reddit users are predominantly male and within the 18‐49 age demographic [35,36]. By country, the United States has the largest number of Reddit users, with other users mostly residing in high-income, English-speaking countries [37]. However, we cannot determine the specific demographics of the users in the Reddit diabetes communities and have assumed that the stated demographics represent the entire Reddit platform.

Given that COVID-19 implications such as number of cases, lockdown policies, and public attitudes toward the pandemic varied by geographical areas, even within the same country, it was beyond the scope of this study to correlate our findings with these factors, as we did not have geographical information of the users.

### Conclusion

#### Themes of Discussion

Using LDA topic modeling from 85,266 posts made between January 2020 and early March 2021 on Reddit forums for people managing T2D, we found 14 topics of discussion that could be assigned into four themes: managing blood glucose, managing lifestyle, coping and receiving support, and obtaining diabetes care. These findings are in line with recommended health behaviors for T2D management that include glycemic control, healthy nutrition, increasing physical activity, reducing sedentary time, and taking prescribed medications. Additionally, discussions encompassed attitudes toward the disease, interactions with family members and caregivers, managing comorbidities, and the sharing of educational resources. These findings validate that Reddit forums for diabetes management not only serve as a support network for people with T2D, but also facilitate discussions that are holistic in nature and extend beyond treating the symptoms of the disease. Among COVID-19–specific posts, topics of discussion included coping with poor mental health, accessing doctor and medications and controlling blood glucose, changing food habits during the pandemic, impact of stress on blood glucose levels, changing status of employment and insurance, and risk of COVID-19 complications

#### Impact on Attitudes Toward T2D Management

The posts classified as COVID-19–related had significantly lower mean sentiment scores than those classified as nonCOVID-19–related, suggesting that the pandemic negatively affected attitudes toward T2D management. Among posts classified as COVID-19–related, topics related to mental health and the impact of stress on glycemic control were associated with lower sentiment scores, whereas posts relating to changing food habits were associated with higher sentiment scores.

#### Significance of Findings

Our study demonstrates that social media analysis is a simpler and effective method for understanding the behaviors and attitudes of people managing chronic diseases such as diabetes, compared to other qualitative inquiry methods. Topics of discussion and sentiment can potentially be generated in real time and over a long period, directly capturing the perspectives of the patient; this is unlike clinical visits, where data is generated from the perspective of the clinician and only presents a snapshot of the patient’s condition. The benefit of social media posts is that they offer infinite possibilities for discussion and provide a more holistic perspective, potentially capturing insights that would have otherwise been overlooked in structured surveys or interviews.

This study has implications on both macro- and microlevels. On a macro scale, extracting data from social media forums can provide an epidemiological overview of behavioral trends of people with T2D, potentially guiding public health policy and formulating treatment guidelines, particularly during public health emergencies such as the COVID-19 pandemic.

## Abbreviations

API: Application Programming Interface
LDA: Latent Dirichlet Allocation
T2D: Type 2 Diabetes
VADER: Valence Aware Dictionary for Sentiment Reasoning
